# Case Report: Spindle cell metaplastic breast carcinoma

**DOI:** 10.3389/fonc.2026.1920800

**Published:** 2026-08-19

**Authors:** Yanze Liu, Xiaoyu Sun, Jiaqi Liu

**Affiliations:** 1 Department of Breast and Thyroid Surgery, Zibo Central Hospital, Zibo, Shandong, China; 2 Department of Pathology, Zibo Central Hospital, Zibo, Shandong, China

**Keywords:** carboplatin, case report, metaplastic breast carcinoma, Miller-Payne grade, neoadjuvant chemotherapy, spindle cell carcinoma, triple-negative breast cancer

## Abstract

**Background:**

Spindle cell metaplastic breast carcinoma (SpCC) is an uncommon subtype of metaplastic breast carcinoma. It often shows a triple-negative phenotype and can closely mimic malignant phyllodes tumor, primary breast sarcoma, and other spindle-cell lesions of the breast.

**Case presentation:**

A 56-year-old woman presented with a 5.4-cm right breast mass. Core needle biopsy showed high-grade spindle cell carcinoma. The tumor expressed CKAE1/AE3, CK8/18, EMA, vimentin, TRPS-1, and partial GATA3, and was negative for ER, PR, HER2, p40, p63, CK5/6, S-100, CD34, and ERG; the Ki-67 index was 85%. BRCA1/2 testing showed no detectable mutation. After six cycles of albumin-bound paclitaxel plus carboplatin, ultrasound and MRI showed marked tumor regression. Modified radical mastectomy revealed residual degenerated malignant cells and ductal carcinoma *in situ*, with Miller-Payne grade 4 response and no metastasis in 17 axillary lymph nodes. The patient received postoperative radiotherapy and low-dose capecitabine. At the latest follow-up on June 25, 2026, there was no evidence of locoregional recurrence or distant metastasis.

**Conclusion:**

This case supports a diagnosis-centered approach to SpCC, using spindle-cell morphology together with epithelial and breast-lineage markers and exclusion of key mimics. A platinum-taxane neoadjuvant regimen achieved a major pathological response in this patient, but evidence for SpCC remains limited and treatment should be individualized.

## Introduction

Metaplastic breast carcinoma (MpBC) is a rare and heterogeneous form of invasive breast cancer in which carcinoma cells show squamous, spindle-cell, chondroid, osseous, or other mesenchymal-like differentiation. In the 5th edition of the World Health Organization (WHO) classification, these tumors are recognized as special-type breast carcinomas with distinct morphology and clinical behavior (1, 2).

The rarity is particularly pronounced for the spindle-cell subtype: recent population-based data and prior reports indicate that breast SpCC accounts for only approximately 0.02%-0.3% of invasive breast carcinomas (3).

Spindle cell metaplastic breast carcinoma (SpCC) is one of the most difficult MpBC patterns to diagnose, especially on core needle biopsy. The differential diagnosis includes malignant phyllodes tumor with stromal overgrowth, primary breast sarcoma, fibromatosis-like metaplastic carcinoma, nodular fasciitis, melanoma, and metastatic sarcomatoid tumors. Because a purely spindle-cell sample may obscure the epithelial nature of the tumor, broad cytokeratin staining, breast-lineage markers, and carefully selected exclusion markers are essential (4, 5).

There is no disease-specific prospective treatment standard for SpCC. Most patients are treated within high-risk breast cancer or triple-negative breast cancer (TNBC) frameworks, although MpBC often responds less well to conventional chemotherapy than ordinary TNBC (6–8). We report a case of high-grade SpCC that showed a marked radiologic and pathological response to albumin-bound paclitaxel plus carboplatin, followed by mastectomy, radiotherapy, and capecitabine.

## Case presentation

### Clinical presentation and imaging findings

A 56-year-old woman noticed a right breast mass approximately one month before presentation. She had no relevant medical history and no known family history of hereditary tumor syndrome. Physical examination revealed a firm, non-tender, poorly mobile mass measuring approximately 6 x 5 cm at the 6 o’clock position of the right breast, 2 cm from the nipple. No nipple discharge or clinically palpable axillary or supraclavicular lymphadenopathy was detected.

**Figure 1:**
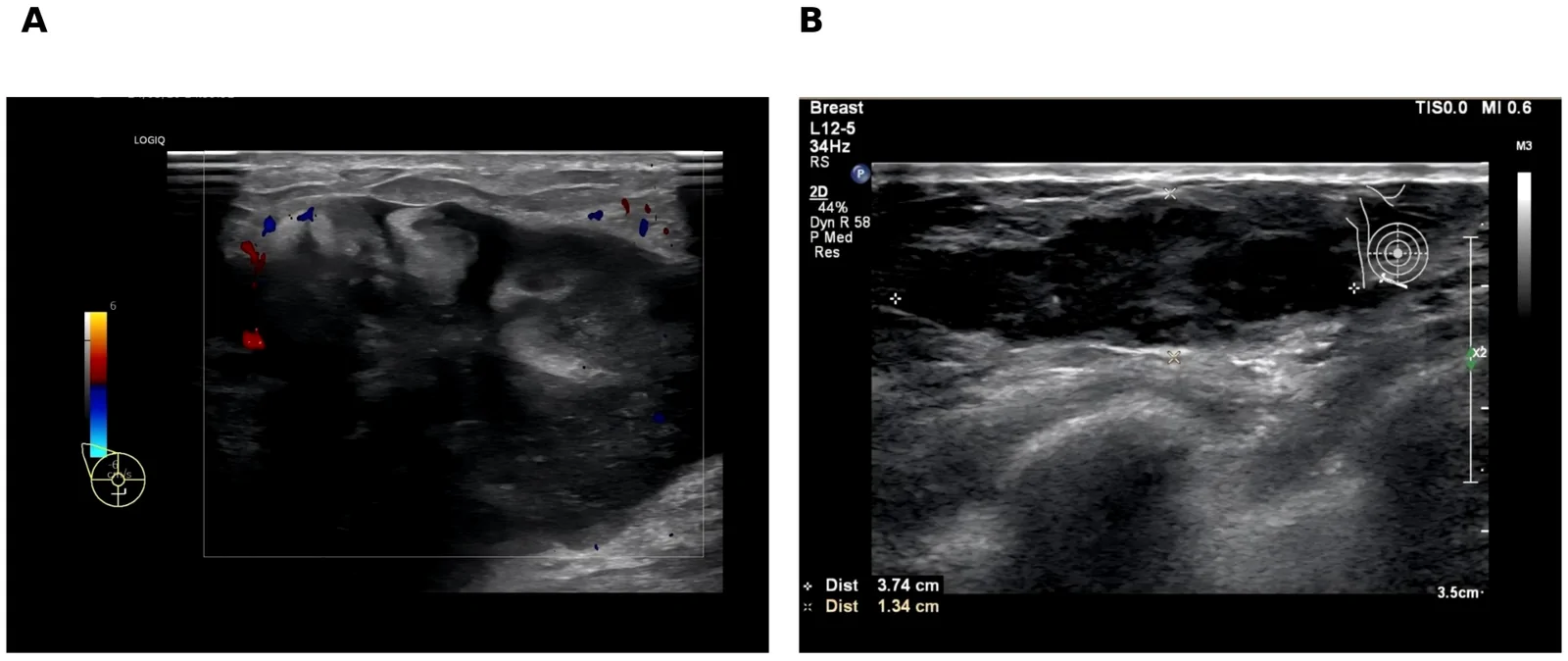
Ultrasonography of the right breast before and after neoadjuvant therapy. **(A)** Baseline color Doppler ultrasonography showing an irregular hypoechoic mass measuring 54 x 32 mm, with heterogeneous echogenicity and intralesional vascular signals; the lesion was classified as BI-RADS 4C. **(B)** Post-neoadjuvant B-mode ultrasonography showing reduction of the residual lesion to 37 x 13 mm; the lesion was classified as BI-RADS 6 because malignancy had been histologically confirmed.

**Figure 2:**
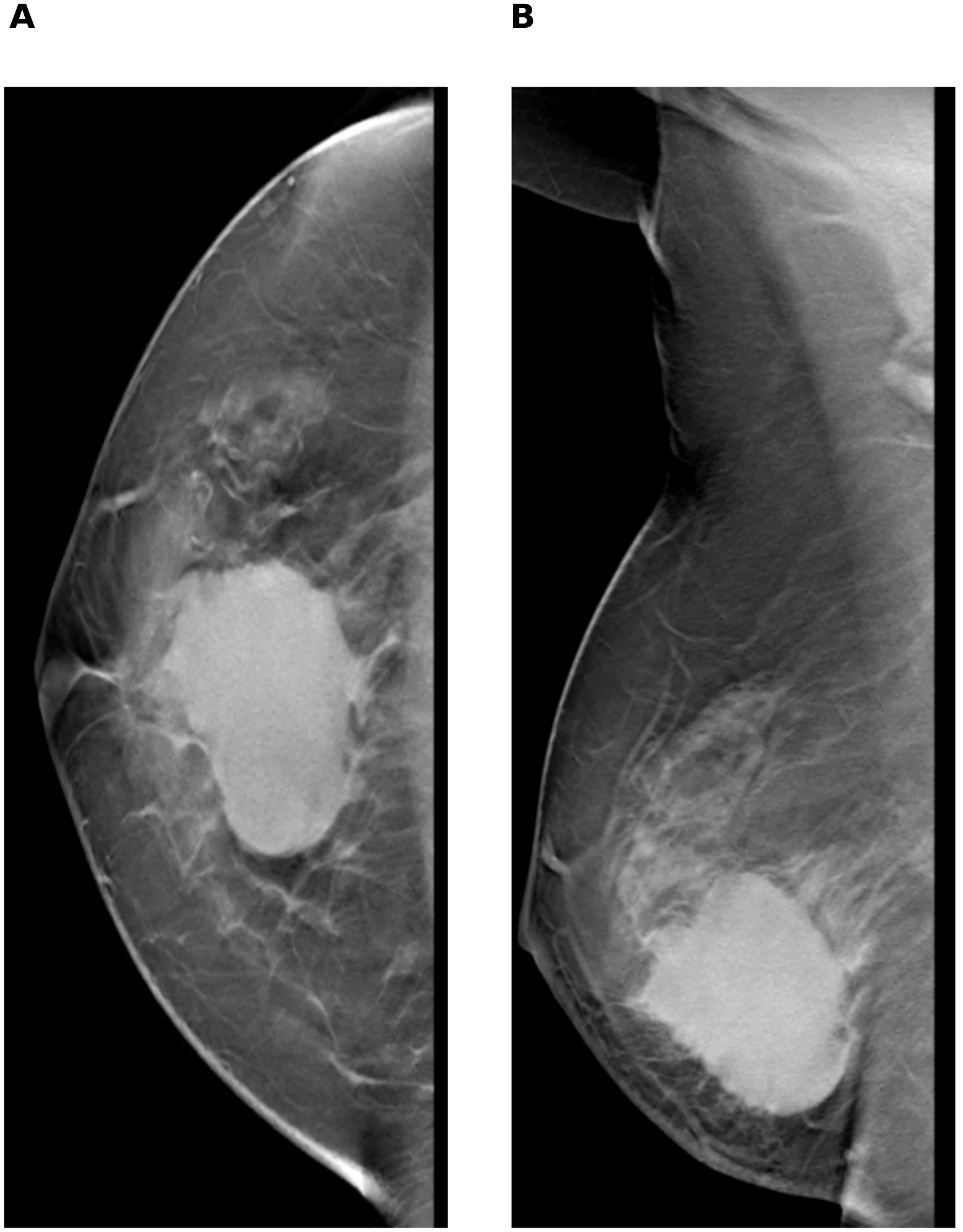
Mammographic images of the right breast before neoadjuvant therapy. **(A)** Craniocaudal view and **(B)** mediolateral oblique view showing a high-density irregular mass measuring approximately 55 x 48 mm in the lower quadrant of the right breast, with spiculated margins, local edema, and disruption of the glandular architecture.

Breast ultrasonography demonstrated an irregular hypoechoic mass measuring 54 x 32 mm in the lower quadrant of the right breast. The lesion showed heterogeneous echogenicity and abundant intralesional blood flow on color Doppler flow imaging. No definite enlarged axillary lymph node was detected by ultrasonography. The lesion was classified as BI-RADS 4C (**Figure 1A**). Mammography revealed a high-density irregular mass measuring approximately 55 x 48 mm in the lower quadrant of the right breast, with spiculated margins, local edema, and disruption of the glandular architecture. A right axillary lymph node measuring approximately 19 x 6.9 mm with an indistinct hilum was observed. The breast lesion was classified as BI-RADS 5 (**Figures 2A, B**).

**Figure 3:**
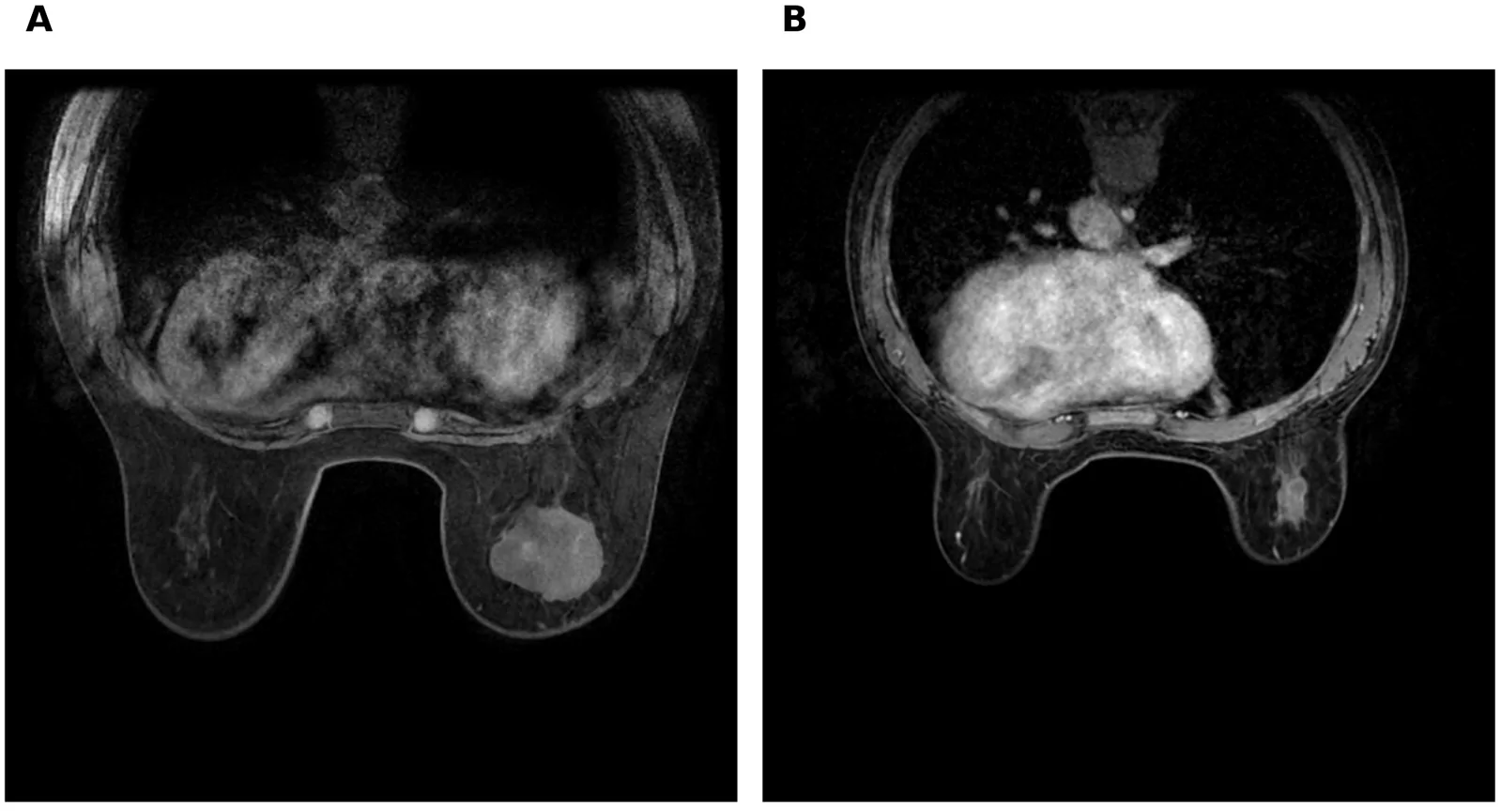
Axial contrast-enhanced breast MRI before and after neoadjuvant therapy. **(A)** Baseline contrast-enhanced MRI showing an irregular mass measuring approximately 5.1 x 4.1 cm with heterogeneous enhancement. **(B)** Post-neoadjuvant contrast-enhanced MRI showing marked reduction of the lesion to approximately 2.4 x 1.2 x 1.7 cm and decreased enhancement. .

Contrast-enhanced breast MRI showed an irregular mass in the lower inner quadrant of the right breast at the 6–8 o’clock position, measuring approximately 5.1 x 4.1 cm (**Figure 3A**). The lesion showed slightly high signal intensity on T2-weighted imaging, low signal intensity on T1-weighted imaging, restricted diffusion on diffusion-weighted imaging, and an apparent diffusion coefficient (ADC) of approximately 0.8-1.1 x 10^-3 mm2/s. Dynamic contrast-enhanced MRI demonstrated heterogeneous enhancement with a plateau-type time-signal intensity curve. The largest right axillary lymph node had a short-axis diameter of approximately 0.8 cm and was considered radiologically suspicious on MRI, although no definite enlarged axillary lymph node was detected by ultrasonography. Because of this discordance between MRI and ultrasonography, and because there was no definite sonographic target, axillary lymph node puncture biopsy was not performed before neoadjuvant therapy. The breast lesion was classified as BI-RADS 5.Based on tumor size and absence of pathologically confirmed nodal metastasis before treatment, the clinical stage was considered at least cT3N0M0, with radiologically suspicious but unconfirmed axillary involvement.

### Core needle biopsy and immunohistochemical profile

**Figure 4:**
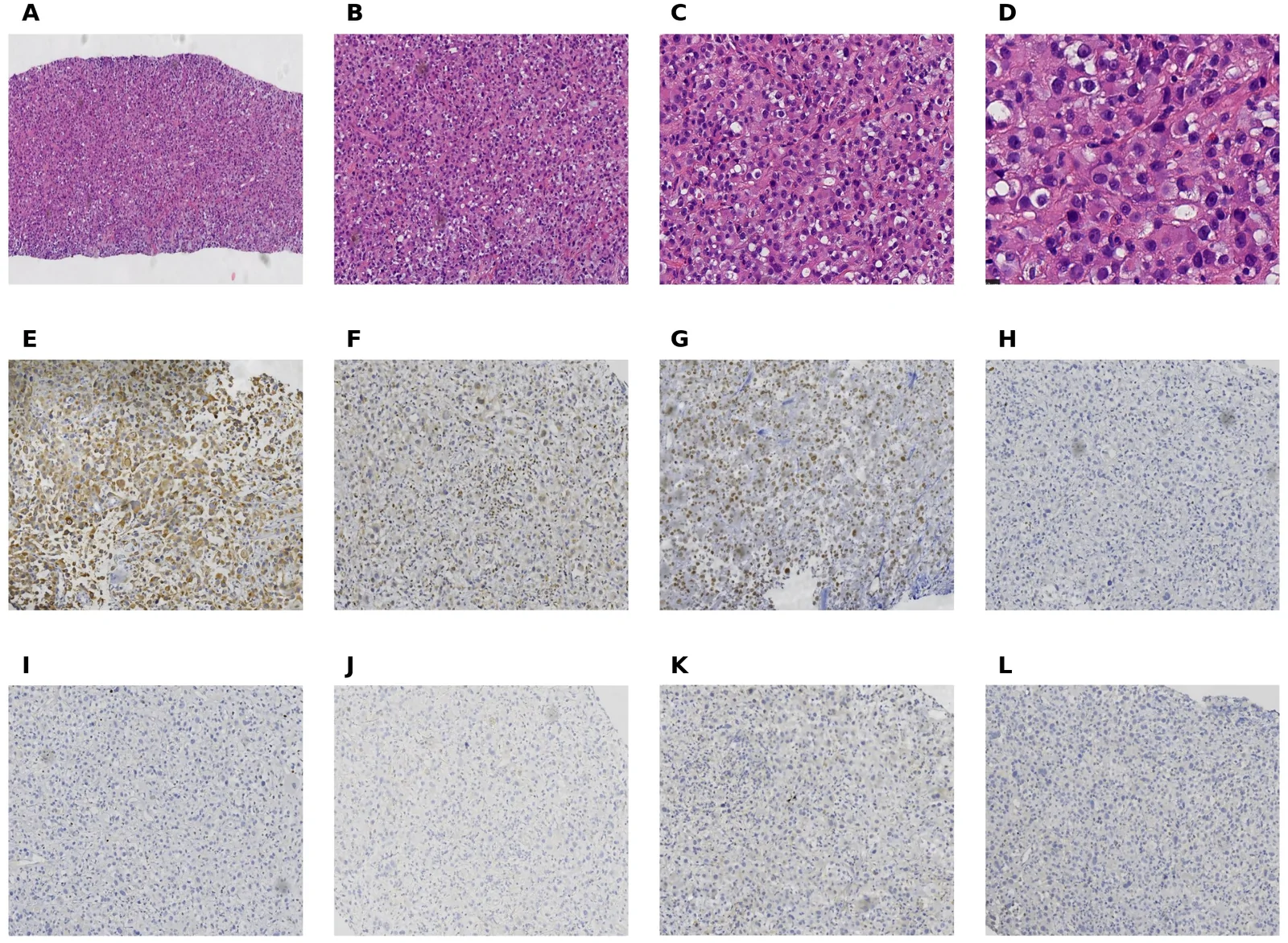
Histological and immunohistochemical features of the core needle biopsy. **(A–D)** H&E-stained sections at 40x, 100x, 200x, and 400x, respectively, showing a high-grade malignant spindle-cell proliferation with fascicular growth, marked nuclear pleomorphism, brisk mitotic activity, and scattered multinucleated tumor giant cells. **(E)** Pan-cytokeratin (AE1/AE3) immunohistochemistry at 100x showing cytoplasmic positivity in tumor cells. **(F)** GATA3 at 100x showing partial nuclear positivity. **(G)** TRPS-1 at 100x showing nuclear positivity. **(H–L)** CK5/6, p63, ER, E-cadherin, and HER2 at 100x, respectively, showing negative staining in tumor cells.

On March 18, 2024, ultrasound-guided core needle biopsy was performed. Histology showed a high-grade malignant spindle-cell tumor with fascicular growth, infiltrative borders, marked cytological atypia, mitotic activity, and focal epithelial differentiation (**Figures 4A–D**). The lesion was diagnosed as poorly differentiated spindle cell carcinoma of the breast.

Immunohistochemistry supported an epithelial malignancy with spindle-cell/metaplastic differentiation. Tumor cells were positive for CKAE1/AE3, CK8/18, EMA, vimentin, TRPS-1, and partially positive for GATA3. CD34 and ERG highlighted vascular structures but were not expressed in tumor cells. The tumor was negative for CK7, S-100, CK5/6, p63, p40, ER, PR, AR, E-cadherin, calretinin, and WT-1. HER2 (clone 4B5) was scored as 0. The Ki-67 index was approximately 85%, and p16 showed moderate positivity in approximately 40% of tumor cells. Pan-cytokeratin (AE1/AE3), GATA3, and TRPS-1 were positive, whereas CK5/6, p63, ER, E-cadherin, and HER2 were negative (**Figures 4E–L**). PD-L1 immunohistochemical testing was not performed.

BRCA1/2 genetic testing was performed, and no BRCA1 or BRCA2 mutation was detected. The integrated diagnostic profile is summarized in **Table 1**.

**Table 1:** Integrated diagnostic profile of the present case.

Diagnostic domain	Findings in the present case	Interpretation
Morphology	High-grade malignant spindle-cell proliferation with fascicular growth, infiltrative borders, marked nuclear pleomorphism, mitotic activity, and focal epithelial differentiation	Supports high-grade spindle cell carcinoma/metaplastic carcinoma rather than a benign spindle-cell lesion
Epithelial differentiation	CKAE1/AE3+, CK8/18+, EMA+	Confirms epithelial carcinoma component and helps exclude primary breast sarcoma
Breast-lineage support	TRPS-1+, partial GATA3+	Supports primary breast origin, although breast-lineage markers may be variable in metaplastic carcinoma
Mesenchymal phenotype	Vimentin+	Consistent with spindle-cell/metaplastic differentiation
Triple-negative phenotype	ER-, PR-, HER2 0; AR-	Consistent with the common immunophenotype of high-grade metaplastic carcinoma
Exclusion markers	S-100-, calretinin-, WT-1-, p40-, p63-, E-cadherin-; CD34/ERG vascular only	Helps exclude melanoma, mesothelial lesions, vascular neoplasm, and some mimics; p63/p40 negativity does not exclude SpCC when broad cytokeratins/EMA are positive
Proliferation and immunotherapy consideration	Ki-67 85%; PD-L1 testing not performed	High proliferative activity; immunotherapy was discussed, but the patient declined treatment
BRCA1/2 genetic testing	No BRCA1 or BRCA2 mutation detected	No BRCA1/2-directed PARP inhibitor strategy was supported by the available genetic result; broader genomic profiling was not performed.

### Neoadjuvant therapy and response assessment

Because of the large tumor size, high-grade morphology, triple-negative phenotype, and high Ki-67 index, neoadjuvant therapy and immunotherapy were recommended after multidisciplinary discussion. Pembrolizumab-based neoadjuvant therapy was discussed with the patient, who declined immunotherapy after counseling regarding its potential benefits and risks. She received six cycles of albumin-bound paclitaxel plus carboplatin.

Post-treatment ultrasonography showed a reduction in tumor size to 37 x 13 mm (**Figure 1B**), with fewer intralesional blood-flow signals on Doppler examination and no definite axillary lymphadenopathy; the lesion was classified as BI-RADS 6 because of the known biopsy-proven malignancy. Post-treatment MRI showed marked reduction of the mass to approximately 2.4 x 1.2 x 1.7 cm, with reduced enhancement compared with baseline (**Figure 3B**). The ADC value increased to approximately 1.1-1.7 x 10^-3 mm2/s, suggesting reduced cellularity after treatment.

### Surgery, postoperative pathology, and adjuvant treatment

On August 13, 2024, after completion of neoadjuvant chemotherapy, the patient underwent modified radical mastectomy. Sentinel lymph node biopsy was not performed. Before treatment, MRI had shown suspicious right axillary lymph nodes, whereas ultrasonography did not identify a definite abnormal node suitable for biopsy. Given the high-grade SpCC diagnosis and the MRI suspicion of nodal disease, the surgical plan was discussed with the patient and family, and axillary lymph node dissection was performed.

Postoperative immunohistochemistry showed only scattered residual epithelial tumor cells. In slice A3, CKAE1/AE3 and CK8/18 highlighted approximately 5% residual tumor cells; ER and PR were negative; p53 was positive in approximately 3% of cells; and the Ki-67 index was approximately 3%. In slice A2, CKAE1/AE3 and CK8/18 were focally positive; ER and PR were negative; p53 was negative; and the Ki-67 index was approximately 5%. The overall treatment response was graded as Miller-Payne grade 4, indicating a major pathological response but not a complete pathological response.

**Figure 5:**
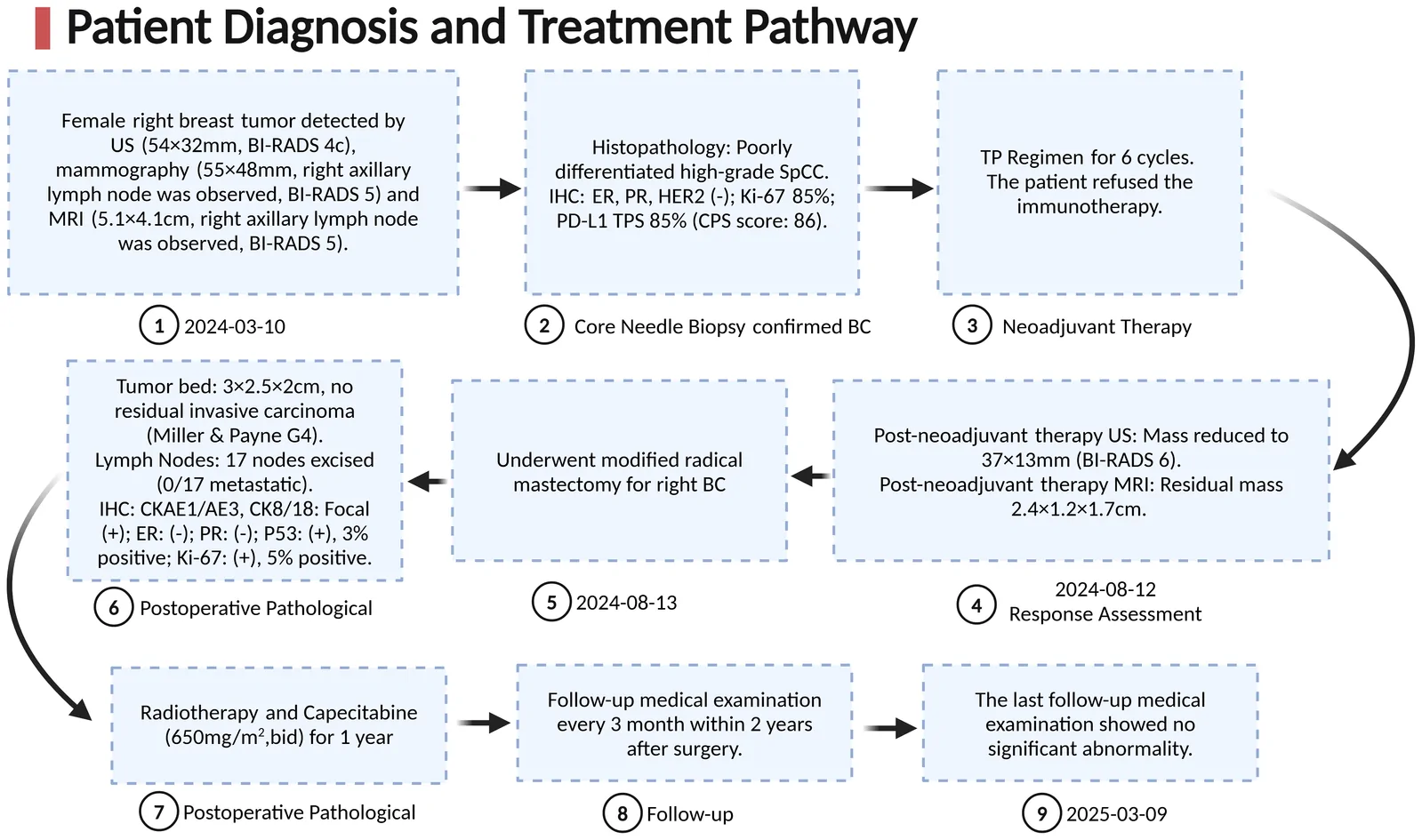
Patient diagnostic and treatment pathway. The flow diagram summarizes initial imaging, core needle biopsy, neoadjuvant albumin-bound paclitaxel plus carboplatin, response assessment, modified radical mastectomy with axillary lymph node dissection, postoperative pathology, adjuvant treatment, and follow-up. US, ultrasound; MG, mammography; MRI, magnetic resonance imaging; SpCC, spindle cell carcinoma; IHC, immunohistochemistry; ER, estrogen receptor; PR, progesterone receptor; HER2, human epidermal growth factor receptor 2; TP regimen, albumin-bound paclitaxel plus carboplatin; M-P, Miller-Payne; ALND, axillary lymph node dissection.

For patients receiving preoperative systemic therapy, the indication and extent of adjuvant radiotherapy were determined according to the maximum pretreatment disease stage together with the pathological findings after systemic therapy. Accordingly, this patient received intensity-modulated radiotherapy (IMRT) to the right chest wall and regional lymphatic drainage areas, including the supraclavicular and infraclavicular regions, axillary lymph nodes, and internal mammary lymph nodes. A conventionally fractionated dose of 50 Gy in 25 fractions (2 Gy per fraction) was delivered once daily, five days per week. Low-dose capecitabine was administered with reference to institutional practice and the 2024 Chinese Society of Clinical Oncology breast cancer guidelines at 650 mg/m² twice daily for one year. At the latest follow-up on June 25, 2026, approximately 22 months after surgery and 27 months after pathological diagnosis, there was no evidence of locoregional recurrence or distant metastasis. The diagnostic and treatment timeline is summarized in **Table 2**, and the overall clinical pathway is illustrated in **Figure 5**.

**Table 2:** Diagnostic and treatment timeline.

Date/period	Clinical event	Key findings	Clinical relevance
March 2024	Initial presentation and imaging	Right breast mass 54 x 32 mm on ultrasound, approximately 55 x 48 mm on mammography, 5.1 x 4.1 cm on MRI; BI-RADS 4C/5	Large high-risk breast malignancy; at least cT3N0M0, with MRI-suspicious but pathologically unconfirmed axillary involvement
March 18, 2024	Core needle biopsy	SpCC; epithelial and breast-lineage markers positive; ER-/PR-/HER2 0; Ki-67 85%; PD-L1 testing not performed	Integrated diagnosis of SpCC; high-risk triple-negative phenotype
Neoadjuvant period	Six cycles of albumin-bound paclitaxel plus carboplatin	Pembrolizumab-based neoadjuvant therapy discussed; patient declined immunotherapy after counseling	TP neoadjuvant chemotherapy selected after MDT discussion
August 2024	Response assessment	Ultrasound: 37 x 13 mm; MRI: 2.4 x 1.2 x 1.7 cm; reduced enhancement and increased ADC	Marked radiological response
August 13, 2024	Modified radical mastectomy with axillary lymph node dissection	Tumor bed 3.0 x 2.5 x 2.0 cm; Miller-Payne grade 4; 0/17 axillary lymph nodes positive; ALND selected after MRI-suspicious nodes, discordant ultrasound findings, aggressive histology, and patient-family discussion	Major pathological response with 0/17 nodes. ALND was an individualized, more extensive approach because pretreatment nodal disease was MRI-suspicious but not biopsy-proven; contemporary guidance would generally favor post-neoadjuvant SLNB in this setting.
Postoperative period	Postoperative radiotherapy and capecitabine	IMRT to the right chest wall and regional lymph nodes (supraclavicular and infraclavicular regions, axillary nodes, and internal mammary nodes), 50 Gy in 25 fractions (2 Gy/fraction), once daily, five days per week; capecitabine 650 mg/m² twice daily for one year	Radiotherapy was planned according to the maximum pretreatment stage and post-treatment pathological findings; capecitabine was selected with reference to the SYSUCC-001 strategy
June 25, 2026	Latest follow-up	No locoregional recurrence or distant metastasis	Approximately 22 months after surgery; continued long-term surveillance recommended

## Discussion

### Diagnostic considerations

The central diagnostic issue in SpCC is whether an apparently mesenchymal spindle-cell tumor still shows convincing epithelial differentiation. This distinction is essential because a similar morphology may occur in primary sarcomas, stromal tumors, and reactive spindle-cell proliferations. In our patient, cytokeratin and EMA expression established the epithelial nature of the lesion, while TRPS-1 and focal GATA3 supported a mammary origin. The infiltrative growth pattern, marked cytological atypia, necrosis, and Ki-67 index of approximately 85% were also in keeping with a high-grade malignant tumor.

No single immunohistochemical marker was decisive. Although p63, p40, and CK5/6 are often useful in metaplastic breast carcinoma, their expression can be patchy or absent; therefore, negative staining does not rule out SpCC. By contrast, CD34 and ERG stained vascular structures but not the tumor cells, making a vascular or stromal spindle-cell neoplasm less likely. The diagnosis was therefore based on the combined morphological and immunophenotypic pattern rather than on one isolated result.

### Differential diagnosis

A practical differential diagnosis begins by confirming epithelial differentiation and then excluding the main spindle-cell mimics. Core biopsy can miss small epithelial foci, so morphology should be interpreted together with a broad cytokeratin panel, breast-lineage markers, and selected exclusion markers (4, 5). In this case, CKAE1/AE3, CK8/18, and EMA supported carcinoma, whereas TRPS-1 and partial GATA3 expression favored a primary breast lesion.

Malignant phyllodes tumor with stromal overgrowth was the most important alternative diagnosis. Leaf-like architecture and a benign epithelial-lined component generally support phyllodes tumor, but both features may be focal and can be missed in a limited core sample (9). Lișcu et al. described this problem directly: a core biopsy suggested a borderline phyllodes tumor, while the resection specimen showed malignancy with a narrow 0.1-cm margin (10). In our patient, no leaf-like pattern or benign epithelial component was identified, and the spindle cells themselves expressed broad epithelial markers, which favored SpCC.

Primary breast sarcoma and angiosarcoma were also considered. However, the tumor-cell expression of cytokeratins and EMA, together with the absence of CD34 and ERG staining in the neoplastic cells, argued against these diagnoses (4, 5). Fibromatosis-like metaplastic carcinoma was less likely because that entity usually shows relatively bland cytology and low proliferative activity, unlike the marked atypia, necrosis, and high Ki-67 index seen here (11, 12).

Reactive spindle-cell lesions, including nodular fasciitis, usually lack epithelial-marker expression and more often show tissue-culture-like fascicles, myxoid stroma, and extravasated erythrocytes (13, 14). Melanoma and metastatic sarcomatoid tumors were also disfavored by the clinical setting, S-100 negativity, and the presence of both epithelial and breast-lineage markers. Taken together, these findings supported a diagnosis of high-grade SpCC.

### Neoadjuvant chemotherapy response

The response of MpBC to neoadjuvant chemotherapy is often limited, although published results are not uniform. Han et al. reported a pathological complete response rate of 17%, whereas Al-Hilli et al. described generally poor responses and occasional progression during treatment (7, 8). More recent series likewise found a pCR rate of only 7.4% and no clear survival advantage for neoadjuvant chemotherapy over primary surgery (15, 16).

Against this background, the Miller-Payne grade 4 response observed in our patient is clinically noteworthy. It should nevertheless be interpreted cautiously, because a single favorable outcome cannot establish the effectiveness of a platinum-taxane regimen in SpCC. The main diagnostic and therapeutic studies relevant to this case are summarized in **Table 3**.

**Table 3:** Selected literature context relevant to the present case.

Topic	Representative literature	Key message	Relevance to present case
Diagnostic approach to spindle-cell breast lesions	Rakha et al. and White et al. diagnostic reviews (4, 5)	Diagnosis requires integration of morphology, cytokeratins, breast-lineage markers, and exclusion markers, particularly on core biopsy	Supports the broad immunohistochemical approach used in this case
WHO classification	WHO 5th edition and related summaries (1, 2)	Spindle cell carcinoma is recognized within metaplastic breast carcinoma	Clarifies terminology and classification
Clinicopathologic features and behavior	Sun et al., Lester et al., and Zhu et al. (3, 6, 19)	SpCC/MpBC commonly shows a triple-negative phenotype, large tumor size, aggressive clinical behavior, and relatively low axillary nodal involvement	Supports cautious prognostic interpretation and the need for long-term follow-up
Neoadjuvant chemotherapy response	Han et al. and Al-Hilli et al. (7, 8)	Published series report low but variable pCR rates to neoadjuvant therapy in MpBC	Makes the present major response to platinum-taxane chemotherapy clinically notable
Immunotherapy evidence in MpBC/TNBC	Immune-checkpoint and anti-PD-1 studies in MpBC/TNBC (20–24)	Immune-checkpoint blockade may be clinically relevant in selected high-risk TNBC/MpBC settings, although evidence in MpBC remains limited and heterogeneous	PD-L1 status was not assessed and the patient declined immunotherapy; therefore, treatment efficacy cannot be inferred
Residual disease and capecitabine	CREATE-X, SYSUCC-001, and CSCO guideline context (25–28)	Capecitabine can be considered for selected high-risk HER2-negative/TNBC patients; low-dose metronomic capecitabine for 1 year is supported by SYSUCC-001 in early TNBC	Supports the rationale for postoperative low-dose capecitabine, while acknowledging that evidence is extrapolated from TNBC rather than SpCC-specific trials
Contemporary systemic therapy and immunotherapy evidence	Chichura et al., Mendes et al., Abuhadra et al., Tan et al., and Schmid et al. (15, 16, 29–31)	Recent data confirm poor and heterogeneous response to conventional neoadjuvant chemotherapy in MpBC, while emerging chemo-immunotherapy data suggest benefit in selected patients but also document progression during treatment.	Places the present major response to platinum-taxane chemotherapy and the declined immunotherapy option in the context of contemporary systemic therapy evidence

Imaging supported the pathological assessment. The lesion decreased from 54 x 32 mm to 37 x 13 mm on ultrasonography and from approximately 5.1 x 4.1 cm to 2.4 x 1.2 x 1.7 cm on MRI. Enhancement also decreased, while ADC values increased. Because MpBC may occasionally progress during systemic treatment, interval imaging is particularly useful for deciding whether chemotherapy should be continued or surgery should be brought forward.

### Axillary management after neoadjuvant therapy

Current international guidance favors less extensive axillary surgery after neoadjuvant treatment whenever oncologically appropriate. For patients who are clinically node-negative before treatment, the Ontario Health/ASCO guideline recommends SLNB at the time of surgery. When nodal metastasis has been confirmed by biopsy and the axilla becomes clinically negative after treatment, retrieval of the clipped node, targeted axillary dissection, or optimized SLNB with dual tracers and removal of at least three sentinel nodes can reduce the false-negative rate. ALND remains appropriate when clinically evident or substantial residual nodal disease persists (17, 18).

This patient did not fit neatly into either category. MRI raised concern for axillary involvement, but physical examination and ultrasonography did not identify a definite abnormal node, and no nodal metastasis was confirmed or clipped before treatment. Her baseline nodal status therefore remained uncertain rather than biopsy-proven cN1. Under current de-escalation principles, post-neoadjuvant SLNB would have been the more guideline-concordant staging procedure. ALND was chosen after multidisciplinary discussion and communication with the patient and family because of the discordant imaging findings, the large high-grade metaplastic tumor, and concern about occult nodal disease. This was an individualized and more extensive approach, not a requirement imposed by SpCC histology. The final result of 0/17 nodes confirms the absence of residual nodal metastasis but cannot determine whether the pretreatment MRI findings represented metastasis or reactive change.

### Immunotherapy considerations

PD-L1 immunohistochemistry was not included in the initial workup because the KEYNOTE-522 regimen for high-risk early TNBC does not use tumor PD-L1 expression as a selection criterion for pembrolizumab-based neoadjuvant therapy (23, 24). PD-L1 testing was therefore not considered necessary for the initial treatment decision. Pembrolizumab was subsequently discussed, but the patient declined immunotherapy after multidisciplinary counseling. This treatment framework is derived mainly from conventional TNBC, and its application to the much rarer MpBC and SpCC populations requires caution.

Early MpBC-specific reports suggest that chemo-immunotherapy may benefit selected patients, but responses remain variable (29, 30). Because immunotherapy was not given in this case, no conclusion can be drawn about its effectiveness, and the tumor’s PD-L1 status remains unknown.

### BRCA1/2 testing and targeted therapy

BRCA1/2 testing was undertaken because the tumor was both triple-negative and high grade. No pathogenic BRCA1 or BRCA2 mutation was identified. The patient therefore did not meet the germline BRCA1/2 requirement for adjuvant olaparib under the OlympiA framework (32). The response to platinum-taxane chemotherapy also cannot be attributed to a documented BRCA-associated homologous-recombination defect. Broader genomic profiling was not performed, so other potentially actionable alterations remain unknown.

### Postoperative treatment

For patients treated with preoperative systemic therapy, postoperative radiotherapy should be based on both the maximum pretreatment disease stage and the pathological findings after systemic therapy. In this case, radiotherapy was selected because the initial tumor was cT3, high grade, triple-negative, and metaplastic, with residual invasive disease after neoadjuvant chemotherapy. Intensity-modulated radiotherapy was delivered to the right chest wall and regional lymphatic drainage areas, including the supraclavicular and infraclavicular regions, axillary lymph nodes, and internal mammary lymph nodes. A conventional schedule of 50 Gy in 25 daily fractions (2 Gy per fraction, five fractions per week) was used.

In SYSUCC-001, metronomic capecitabine at 650 mg/m2 twice daily for one year improved 5-year disease-free survival after standard treatment for operable TNBC (26), and the updated analysis suggested that this benefit was sustained over longer follow-up (27). We used the same schedule because the patient had several high-risk features, including a large initial tumor, a triple-negative phenotype, a high Ki-67 index, metaplastic histology, and residual tumor cells after neoadjuvant therapy. The choice was individualized and made within a high-risk TNBC framework, with reference to the 2024 CSCO guideline (28). Evidence specific to SpCC remains lacking.

### Limitations

This report has several limitations. First, it describes only one patient, so the marked response cannot be used to infer the general effectiveness of platinum-taxane chemotherapy in SpCC. Follow-up has reached approximately 22 months after surgery, but this interval is still too short to assess long-term disease control in a rare and potentially aggressive tumor.

Second, PD-L1 testing and broad next-generation sequencing were not performed, which limits the immune and molecular characterization of the tumor. The patient also declined immunotherapy, so its clinical effect could not be assessed. Finally, pretreatment axillary staging was incomplete because the imaging findings were discordant and no suspicious node was biopsied or clipped. As a result, the true baseline nodal status remains uncertain, and the decision to perform ALND was more extensive than the de-escalated SLNB approach generally recommended when nodal metastasis has not been pathologically confirmed.

## Conclusion

SpCC of the breast is rare and easily confused with other spindle-cell lesions. Diagnosis should be based on morphology, epithelial marker expression, breast-lineage markers, and exclusion of key mimics. In this patient, albumin-bound paclitaxel plus carboplatin produced marked radiologic regression and a Miller-Payne grade 4 pathological response, followed by surgery, radiotherapy, and capecitabine. The absence of recurrence or metastasis at 22 months after surgery is encouraging, but longer follow-up and additional cases are needed before drawing treatment conclusions.

## Data Availability

The raw data supporting the conclusions of this article will be made available by the authors, without undue reservation.

## References

[1] WHO Classification of Tumours Editorial Board . Breast Tumours. 5th ed Vol. 2. . Lyon: International Agency for Research on Cancer (2019).

[2] Tan PH Ellis I Allison K Brogi E Fox SB Lakhani S . The 2019 World Health Organization classification of tumours of the breast. Histopathology. (2020) 77:181–5. doi: 10.1111/his.14091 32056259

[3] Sun Y Chen H Wang K Liu Y . Clinicopathological characteristics and survival outcomes in spindle cell carcinoma (SpCC) of the breast: a SEER population-based study. Med (Baltimore). (2025) 104:e44851. doi: 10.1097/MD.0000000000044851 41054106 PMC12499666

[4] Rakha EA Brogi E Castellano I Quinn C . Spindle cell lesions of the breast: a diagnostic approach. Virchows Arch. (2022) 480:127–45. doi: 10.1007/s00428-021-03162-x 34322734 PMC8983634

[5] White MJ Cimino-Mathews A . Diagnostic approach to mesenchymal and spindle cell tumors of the breast. Adv Anat Pathol. (2024) 31:411–28. doi: 10.1097/PAP.0000000000000464 39466698

[6] Lester TR Hunt KK Nayeemuddin KM Bassett RL Gonzalez-Angulo AM Feig BW . Metaplastic sarcomatoid carcinoma of the breast appears more aggressive than other triple receptor-negative breast cancers. Breast Cancer Res Treat. (2012) 131:41–8. doi: 10.1007/s10549-011-1393-6 21331622 PMC3867807

[7] Han M Salamat A Zhu L Zhang H Clark BZ Dabbs DJ . Metaplastic breast carcinoma: a clinical-pathologic study of 97 cases with subset analysis of response to neoadjuvant chemotherapy. Mod Pathol. (2019) 32:807–16. doi: 10.1038/s41379-019-0208-x 30723293

[8] Al-Hilli Z Choong G Keeney MG Visscher DW Ingle JN Goetz MP . Metaplastic breast cancer has a poor response to neoadjuvant systemic therapy. Breast Cancer Res Treat. (2019) 176:709–16. doi: 10.1007/s10549-019-05264-2 31119569 PMC7469521

[9] Lissidini G Mule A Santoro A Papa G Nicosia L Cassano E . Malignant phyllodes tumor of the breast: a systematic review. Pathologica. (2022) 114:111–20. doi: 10.32074/1591-951X-754 35414723 PMC9248247

[10] Lișcu HD Ionescu AI Mologani I Verga N . Phyllodes tumor of the breast: a case report regarding the importance of fast interdisciplinary management. Rep (Basel). (2025) 8:17. doi: 10.3390/reports8010017 40729230 PMC12199950

[11] Sneige N Yaziji H Mandavilli SR Perez ER Ordonez NG Gown AM . Low-grade (fibromatosis-like) spindle cell carcinoma of the breast. Am J Surg Pathol. (2001) 25:1009–16. doi: 10.1097/00000478-200108000-00004 11474284

[12] Victoor J Bourgain C Vander Borght S Vanden Bempt I De Rop C Floris G . Fibromatosis-like metaplastic carcinoma: a case report and review of the literature. Diagn Pathol. (2020) 15:20. doi: 10.1186/s13000-020-00943-x 32127014 PMC7053053

[13] East EG Carter CS Sciallis AP . Cellular spindled histiocytic pseudotumor: a benign mimic of spindle cell neoplasia of the breast. Arch Pathol Lab Med. (2019) 143:1497–503. doi: 10.5858/arpa.2019-0421-RA 31765251

[14] Allison DB Wakely PE Siddiqui MT Ali SZ . Nodular fasciitis: a frequent diagnostic pitfall on fine-needle aspiration. Cancer Cytopathol. (2017) 125:20–9. doi: 10.1002/cncy.21768 27525591

[15] Chichura AM Chen X Nelis M Kopicky L Wei W Ali A . Neoadjuvant chemotherapy is associated with worse 5-year overall survival in patients with metaplastic breast cancer compared with primary surgery: a National Cancer Database analysis. Ann Surg Oncol. (2025) 32:8525–33. doi: 10.1245/s10434-025-18085-z 40833542 PMC12494608

[16] Mendes TAR de Oliveira-Junior I Brenelli FP Cardoso-Filho C Zeferino LC . Clinicopathological characteristics, treatments and oncological outcomes in metaplastic breast cancer: a Brazilian multicenter analysis. Front Oncol. (2025) 15:1568178. doi: 10.3389/fonc.2025.1568178 41089505 PMC12515624

[17] Brackstone M Baldassarre FG Perera FE Cil T Chavez Mac Gregor M Dayes IS . Management of the axilla in early-stage breast cancer: Ontario Health (Cancer Care Ontario) and ASCO guideline. J Clin Oncol. (2021) 39:3056–82. doi: 10.1200/JCO.21.00934 34279999

[18] Reimer T Kuehn T Mueller V Ditsch N Fehm T Albert US . AGO Breast Commission recommendations for the surgical therapy of breast cancer: Working Group on Gynecologic Cancers (AGO) update 2025. Eur J Surg Oncol. (2025) 51:110445. doi: 10.1016/j.ejso.2025.110445 40983010

[19] Zhu H Li K Dong DD Fu J Liu DD Wang L . Spindle cell metaplastic carcinoma of breast: a clinicopathological and immunohistochemical analysis. Asia Pac J Clin Oncol. (2017) 13:e72–8. doi: 10.1111/ajco.12322 25483573

[20] Tray N Taff J Singh B Suh J Ngo N Kwa M . Metaplastic breast cancers: genomic profiling, mutational burden and tumor-infiltrating lymphocytes. Breast. (2019) 44:29–32. doi: 10.1016/j.breast.2018.12.010 30609392

[21] Kalaw E Lim M Kutasovic JR Sokolova A Taege L Johnstone KJ . Metaplastic breast cancers frequently express immune checkpoint markers FOXP3 and PD-L1. Br J Cancer. (2020) 123:1665–72. doi: 10.1038/s41416-020-01065-3 32939056 PMC7686342

[22] Kim I Rajamanickam V Bernard B Chun B Wu Y Martel M . A case series of metastatic metaplastic breast carcinoma treated with anti-PD-1 therapy. Front Oncol. (2021) 11:635237. doi: 10.3389/fonc.2021.635237 34168978 PMC8217650

[23] Schmid P Cortes J Pusztai L McArthur H Kümmel S Bergh J . Pembrolizumab for early triple-negative breast cancer. N Engl J Med. (2020) 382:810–21. doi: 10.1056/NEJMoa1910549 32101663

[24] Schmid P Cortes J Dent R Pusztai L McArthur H Kümmel S . Event-free survival with pembrolizumab in early triple-negative breast cancer. N Engl J Med. (2022) 386:556–67. doi: 10.1056/NEJMoa2112651 35139274

[25] Masuda N Lee SJ Ohtani S Im YH Lee ES Yokota I . Adjuvant capecitabine for breast cancer after preoperative chemotherapy. N Engl J Med. (2017) 376:2147–59. doi: 10.1056/NEJMoa1612645 28564564

[26] Wang X Wang SS Huang H Cai L Zhao L Peng RJ . Effect of capecitabine maintenance therapy using lower dosage and higher frequency vs observation on disease-free survival among patients with early-stage triple-negative breast cancer who had received standard treatment: the SYSUCC-001 randomized clinical trial. JAMA. (2021) 325:50–8. doi: 10.1001/jama.2020.23370 33300950 PMC7729589

[27] Yuan J Bi XW Hua X Huang H Cai L Zhao L . Metronomic capecitabine as extended adjuvant chemotherapy for early triple-negative breast cancer (SYSUCC-001): updated 10-year outcomes and post-hoc exploratory biomarker analysis from a randomised, phase 3 trial. Lancet Oncol. (2025) 26:1575–83. doi: 10.1016/S1470-2045(25)00545-5 41308674

[28] Li J Hao C Wang K Zhang J Chen J Liu Y . Chinese society of clinical oncology (CSCO) breast cancer guidelines 2024. Transl Breast Cancer Res. (2024) 5:18. doi: 10.21037/tbcr-24-31 39184927 PMC11341997

[29] Abuhadra N Pareja F White C Chen Y Wen H Dikoglu E . Predictors of response to neoadjuvant chemo-immunotherapy in metaplastic triple-negative breast cancer. NPJ Breast Cancer. (2025) 11:110. doi: 10.1038/s41523-025-00816-w 41038871 PMC12491465

[30] Tan Q Li N Wang Y Du T Liang G Zhao Z . Promising response to neoadjuvant chemotherapy plus immunotherapy in metaplastic breast carcinoma. Breast Cancer (Dove Med Press). (2025) 17:447–54. doi: 10.2147/BCTT.S512790 40433440 PMC12109030

[31] Schmid P Cortes J Dent R McArthur H Pusztai L Kümmel S . Overall survival with pembrolizumab in early-stage triple-negative breast cancer. N Engl J Med. (2024) 391:1981–91. doi: 10.1056/NEJMoa2409932 39282906

[32] Tutt ANJ Garber JE Kaufman B Viale G Fumagalli D Rastogi P . Adjuvant olaparib for patients with BRCA1- or BRCA2-mutated breast cancer. N Engl J Med. (2021) 384:2394–405. doi: 10.1056/NEJMoa2105215 34081848 PMC9126186

